# Dynamic architecture of mammalian paternal chromatin: histone-to-protamine exchange and post-fertilization reprogramming

**DOI:** 10.1186/s13072-025-00651-0

**Published:** 2025-12-07

**Authors:** Amir Masoud Firouzabadi, Farzaneh Fesahat, Seyed Morteza Seifati

**Affiliations:** 1 Department of Biology, Ashk.C., Islamic Azad University, Ashkezar, Iran; 2 Medical Biotechnology Research Center, Ashk.C., Islamic Azad University, Ashkezar, Iran; 3 Reproductive Immunology Research Center, Shahid Sadoughi University of Medical Sciences, Yazd, Iran

**Keywords:** Chromatin, Protamine, Histone, Epigenetic reprogramming, Sperm, DNA packaging

## Abstract

Chromatin remodeling in male germ cells and after fertilization plays a pivotal role in genetic transmission and early embryonic development. During spermatogenesis, histone-based chromatin undergoes progressive reorganization: canonical histones are gradually replaced by testis-specific variants, then by transition proteins, and ultimately by highly basic protamines (PRM1 and PRM2). This hierarchical replacement, modulated by histone post-translational modifications—including hyperacetylation, ubiquitination, and dynamic methylation—and supported by molecular chaperones and chromatin remodelers, ensures the efficient compaction of paternal DNA required for sperm function and genome stability. Upon fertilization, paternal chromatin undergoes rapid decondensation as protamine disulfide bonds are reduced, allowing maternal histone incorporation. In parallel, the paternal genome experiences extensive but regulated epigenetic reprogramming, including DNA demethylation and histone modification changes, which together establish a transcriptionally permissive state for zygotic genome activation and maternal–paternal chromatin integration. This review aims to provide an overview of chromatin remodeling from the male germline to post-fertilization stages in mammals, integrating recent findings on the molecular machinery involved in histone-to-protamine replacement and its reversal during early embryogenesis. It outlines the major processes involved in histone-to-protamine exchange, protamine removal, and chromatin reorganization after fertilization, defining the scope of the review for readers. Where available, comparative data from vertebrate and invertebrate models are discussed to provide an initial perspective on the possible evolutionary conservation of these mechanisms. Clarifying these processes offers valuable insight into male fertility, early embryonic regulation, and potential epigenetic inheritance, with implications for both fundamental and applied reproductive biology.

## Introduction

The successful transmission of genetic information is fundamentally dependent on the precise structural organization of the paternal genome, a requirement that is indispensable for both successful fertilization and the initial cascade of embryonic development [1]. Within eukaryotic nuclei, the immense length of DNA is managed by its intricate folding around histone protein octamers, culminating in the formation of nucleosomes, which are the fundamental organizational units of chromatin. This complex, hierarchical packaging system does more than simply condense the DNA; it establishes a versatile platform for modulating DNA accessibility, which is a prerequisite for crucial cellular events like gene transcription and DNA replication [2].

The dynamic nature of this chromatin structure is governed by an array of post-translational modifications (PTMs) on the histone tails, achieved through the addition of distinct chemical moieties such as acetyl or methyl groups at specific regulatory positions. These modifications serve a dual purpose: they directly modify the electrostatic interaction between the histones and the DNA helix, and they function as “landing pads” for the recruitment of specialized effector proteins that execute DNA-dependent processes [3, 4]. The specific deployment of the enzymes that catalyze these histone modifications is a tightly controlled process. These modifying enzymes are strategically targeted, either through their affinity for particular DNA sequences or via interactions with other DNA-binding proteins [5]. For instance, sequence-specific transcription factors are critical recruiters, bringing histone modifiers directly to gene promoter regions to modulate transcriptional output. Furthermore, the core transcription machinery itself facilitates the localized modification of histones along the active gene bodies as transcription progresses through the coding sequence [6].

Chromatin is also profoundly shaped by DNA replication [7]. During cell division, for two daughter cells to inherit equivalent genetic information, the genome must be fully and accurately duplicated in S phase. This involves the use of numerous replication initiation sites, of which only a subset fires stochastically [8, 9]. Specific histone modifiers are recruited to the replication machinery to modify histones at replication origins. Furthermore, as replication progresses, parental histones are ejected and new histones are synthesized for wrapping DNA Ensuring a full nucleosome complement on newly synthesized DNA requires the concerted recycling of parental and deposition of de novo synthesized histones. This mixing with new histones dilutes parental histones and their associated marks [10–13]. To restore pre-existing PTMs on chromatin, read-write mechanisms have been proposed to mark new histones based on information from the old ones. Thus, the mechanisms regulating DNA replication interplay with the recovery of epigenetic states, which can itself impact DNA replication [14–16]. The patterns of histone modifications therefore integrate the action of different DNA-related processes, in particular gene expression and DNA replication. For example, H3K4me3 and H3K9ac correlate with gene expression, while H3K27me3 is found primarily in repressive regions [3, 17, 18]. The complex interplay of these histone modifiers and the mechanisms of mark inheritance provides the foundational, flexible epigenetic machinery. In the context of the male germline, this machinery is specifically co-opted and repurposed to facilitate the unprecedented, large-scale chromatin reorganization required to transition the genome from an actively transcribing state to one of ultra-stable, silent compaction [19, 20].

It is critical to note that the infertility rate of couples has increased to 12%–18% worldwide, with male factors contributing to 40%−50% of this disease. This involves transitioning from a histone-based nucleosomal structure, typical of somatic cells, to an extremely condensed protamine-based structure in mature sperm [21, 145]. This hyper-compaction is crucial for reducing the sperm head volume, enabling efficient motility, and protecting the paternal DNA from various environmental insults encountered during its journey to the oocyte [22, 23, 148]. Following fertilization, this highly condensed paternal chromatin must rapidly decondense and be reprogrammed to integrate with the maternal genome and facilitate zygotic gene activation [24, 147]. The precise regulation of spermatogenesis relies on synergistic interactions between genetic and epigenetic factors, which include DNA methylation, histone modifications, Chromatin Remodeling Complexes (CRCs), and non-coding RNAs [25]. This review aims to integrate recent findings on the molecular machinery of histone-to-protamine replacement and its reversal during early embryogenesis.

## Chromatin architecture: fundamental differences between sperm and somatic cells

In mature mammalian sperm, the structure and protein components responsible for chromatin packaging are fundamentally distinct from those found in somatic cells. In somatic cells, chromatin is primarily organized by a group of basic proteins called histones. These include core histones (H2A, H2B, H3, H4), which form an octamer around which DNA wraps, and linker histone (H1), which helps stabilize the structure [26, 27]. The repeating unit of this structure is called a nucleosome, giving a “beads-on-a-string” appearance [28, 29]. In mature sperm, however, histones are largely replaced by smaller, more basic proteins called protamines [primarily Protamine 1 and 2 (PRM1 and 2)], as well as by transition proteins (TNP), which act during the intermediate phases of this histone replacement. Mutations in either PRM1 and PRM2 disrupt normal sperm chromatin formation, which impairs sperm function [30]. Such alterations are also strongly correlated with male infertility, including non-obstructive azoospermia (NOA), due to abnormal protamine ratios (especially PRM1/PRM2 imbalance) and defective chromatin condensation [25].

Notably, a small fraction of histones—ranging from approximately 10%–15% in humans and 1%–8% in mice, with an overall variation of 1–15% across mammalian species—are retained in the mature sperm chromatin, forming a heterogeneous mixture of nucleo-histones and nucleo-protamines [31–34]. These retained histones are not randomly distributed but are preferentially localized to specific genomic regions, including gene promoters, telomeres, repetitive elements, and CpG-rich loci—particularly those associated with developmental and regulatory genes [32, 33, 35]. This selective retention highlights the sophisticated “dual mandate” of sperm chromatin remodeling. If protection and compaction were the sole objectives, a complete and uniform histone replacement would be the most logical strategy. The fact that specific nucleosomes are preserved strongly implies that the sperm genome is not a “blank” sequence. Instead, this retention acts as a critical mechanism of transgenerational epigenetic inheritance, carrying a “bookmark” or “poised” state at key developmental genes, preparing them for rapid activation after fertilization [36]. This selective retention, together with distinct histone variants and bivalent modifications such as H3K4me3 and H3K27me3, implies a potential role of sperm-retained histones in marking developmentally important genes for early embryonic activation and in mediating transgenerational epigenetic inheritance [32, 33]. These bivalent marks (active H3K4me3 and repressive H3K27me3) are critical for maintaining the poised state of developmental genes, marking them for early embryonic activation and ensuring proper epigenetic inheritance across generations [25].

While the nucleosomal structure in somatic cells leads to chromatin fibers with varying degrees of compaction, the incorporation of protamines in sperm causes DNA to condense into extremely compact toroidal structures and hexagonal arrays [31, 37, 38]. The resulting 3D organization of sperm chromatin involves the formation of toroidal structures and nuclear halos. This unique structure can be visualized using techniques like Hi-C and is fundamentally different from the chromatin organization in species like zebrafish, which lack protamines entirely [31, 37, 38]. The resulting difference in compaction is striking—sperm chromatin is about ten times denser than somatic chromatin, which in differentiated somatic cells comprises a mixture of euchromatic and heterochromatic regions depending on the cell type. This high degree of compaction renders the paternal genome transcriptionally and replicative inactive, providing physical protection against nucleases and mechanical stress during its delivery to the oocyte [39–41] (Table 1). This extensive remodeling is not merely a structural formality; it is an evolutionary solution to several critical biological challenges. The sperm’s journey from the male epididymis through the female reproductive tract is perilous, exposing the genome to significant mechanical stress, oxidative damage, and enzymatic threats, including nucleases. The relatively open and fragile “beads-on-a-string” structure of a somatic cell is entirely unfit for this task. The near-crystalline density of protamine-bound chromatin provides an unparalleled level of physical “armor” to ensure the paternal DNA’s integrity. Furthermore, this transcriptionally inert state is a functional imperative. The sperm is a highly specialized delivery vehicle whose finite energy reserves must be allocated entirely to motility, not to wasteful gene expression. This genomic “shutdown” also serves a crucial developmental purpose: it ensures that upon fertilization, the oocyte’s cytoplasmic factors, and not the paternal genome, gain complete control over the initiation of the embryonic transcriptional program [42].

**Table 1:** Comparison of chromatin packaging in sperm and somatic cells

Feature	Sperm	Somatic cells	References
Main proteins	Transition proteins (TNP1, TNP2) protamines (PRM1, PRM2)	Core histones (H2A, H2B, H3, H4), linker histone (H1)	[26, 31]
Chromatin structure	DNA-protamine toroids; DNA is tightly packed in toroid arrays	DNA wrapped around histone octamers (nucleosomes)	[28, 31]
Level of compaction	Very high compaction (about 6–10 times more than somatic cells)	Moderate compaction (about 10 times less than sperm)	[31].
Supercoiling	Resistant to DNase I, micrococcal nuclease, and physical stress	Sensitive to DNase I, micrococcal nuclease	[39]
DNA activity	Transcription and replication stop; DNA is protected during epididymal transit	Transcription, replication, DNA repair possible	[39]
DNA damage sensitivity	Highly resistant due to high compaction and protamine protection	Relatively higher sensitivity due to chromatin accessibility	[39]

## Histone replacement in spermatids

During spermiogenesis, the replacement of canonical histones with transition proteins and ultimately protamines is orchestrated through two major, interrelated mechanisms that promote chromatin remodeling and compaction:

- Incorporation of Testis-Specific Histone Variants: Certain canonical histones are replaced by testis-specific variants that have altered structural properties, resulting in weaker interactions with DNA. This reduced affinity facilitates their subsequent removal during chromatin reorganization [43].
- PTMs of Remaining Histones: Histones that are not initially replaced undergo specific PTMs—particularly acetylation and ubiquitination of histone H4—which destabilize nucleosomes and increase chromatin accessibility, thereby making histone eviction more efficient [43].

Together, these mechanisms create a permissive chromatin environment that allows for the orderly incorporation of TNP1 and TNP2 and the eventual replacement by PRM1 and PRM2, culminating in the formation of a highly compacted and transcriptionally silent sperm genome.

### Incorporation of testis-specific histone variants

A pivotal step in the chromatin remodeling that occurs during spermiogenesis is the incorporation of testis-specific histone variants. These variants, which differ structurally and functionally from canonical histones, are expressed independently of DNA replication and are specifically enriched during post-meiotic stages of germ cell development [44, 45]. Their incorporation weakens histone-DNA interactions, thereby increasing chromatin accessibility and facilitating subsequent histone displacement. This process creates a chromatin landscape that is permissive for the stepwise replacement of histones by transition proteins and, ultimately, protamines. These variants act as molecular intermediates, priming the genome for the dramatic compaction required in mature sperm [46, 47].

#### H1 variants

H1 play a crucial role in promoting and stabilizing higher-order chromatin structures [48]. Among the 11 subtypes of histone H1 identified in mammals, H1T, H1T2, and HILS1 are testis-specific [49]. H1T is expressed from mid- to late pachytene spermatocytes and remains abundant in elongating spermatids [50, 51]. Unlike somatic H1 variants, H1T has a lower binding affinity for nucleosomes, suggesting it promotes a relatively open chromatin structure that supports histone removal [51, 52]. Interestingly, mice lacking H1T are fertile and show no overt defects in spermatogenesis, indicating functional redundancy with other H1 variants [51, 53]. H1T2 is specifically localized to the apical pole of round and elongating spermatid nuclei, absent in mature spermatozoa, and is essential for spermiogenesis; its deletion causes impaired nuclear condensation, abnormal spermatid elongation, and infertility [54, 55]. HILS1, another testis-specific variant, is expressed in elongating and elongated spermatids. As the least conserved H1 variant, it poorly compacts chromatin and is thought to contribute uniquely to the histone-to-protamine transition [56, 57]. Although related proteins in Drosophila are known to influence nuclear condensation, the specific roles of HILS1 in mammals remain to be clarified.

#### H2A variants

Several testis-specific H2A variants—TH2A, H2AL1, H2AL2, H2AL3, and H2A.B—have been identified [58, 59]. TH2A is present in early primary spermatocytes and disappears as nuclear condensation proceeds [60]. It contributes to chromatin decondensation by weakening histone-DNA interactions [61, 62]. Although Th2a knockout mice have not been reported, dual knockout of Th2a and Th2b impairs chromatin incorporation of TNP2, causes elevated H2B levels, and leads to infertility [63]. H2AL2 is specifically expressed in condensing spermatids, coinciding with *TNP* expression. It is essential for loading TNPs onto nucleosomes and efficient protamine replacement [43, 64]. H2A.B is expressed from the pachytene stage through to round spermatids and promotes chromatin decondensation [59, 65]. In H2a.b-null mice, defective TNP1-to-protamine transition and H2AL2 mislocalization result in subfertility and aberrant sperm morphology [66].

#### H2B variants

TH2B is a well-studied testis-specific H2B variant, replacing somatic H2B during meiosis and persisting through spermatid elongation [67, 68]. Structural analyses suggest it destabilizes nucleosomes and facilitates histone removal [69]. Despite the severe infertility seen with dominant-negative TH2B mutants, Th2b-null mice are fertile, likely due to compensatory upregulation of somatic H2B and altered methylation patterns on H2B and H4 [70, 71]. In humans, polymorphisms in the testis-specific variant H2BFWT are associated with infertility [60, 72, 73]. Additional variants such as ssH2B and H2BL1 are enriched in spermatids, although their precise functions remain to be defined [74].

#### H3 variants

Beyond canonical H3.1 and H3.2, three H3 variants—H3.3, H3T (H3.4), and H3.5—are expressed in testes [21, 75]. H3.3 supports an open chromatin structure and transcriptional activity and is required for proper TNP1 and PRM1 deposition [76, 77]. H3T, specific to spermatocytes, promotes chromatin flexibility; its deletion leads to azoospermia and male infertility [78]. H3.5, predominantly found in human spermatogonia and spermatocytes, is reduced in non-obstructive azoospermia, though its function remains unclear [79].

#### Functional role of histone chaperones in variant exchange during spermiogenesis

Following the incorporation of testis-specific histone variants into spermatid chromatin, a critical step in chromatin remodeling involves the action of histone chaperones. These chaperones transiently assist in the deposition and removal of histone variants, thereby reducing nucleosome stability and enabling the stepwise replacement of histones with transition proteins and, ultimately, protamines [80].

Two major histone chaperones, Anti-Silencing Function 1 A (ASF1A) and Nucleoplasmin 2 (NPM2), have been identified as essential regulators of this process. ASF1A plays a key role in the incorporation and stabilization of histone variant H3.3, which is typically enriched at transcriptionally active genomic regions and exhibits greater structural flexibility than canonical H3. The targeted deposition of H3.3 promotes a gradual release of DNA from nucleosomes, thereby facilitating chromatin relaxation and preparing the genome for the binding of transition proteins [81].

In parallel, NPM2 is critically involved in the exchange of TH2A and TH2B, two testis-specific variants of H2A and H2B. These variants contribute to nucleosome destabilization and chromatin loosening, essential for the next phase of compaction. NPM2 mediates the removal of these histones and primes the chromatin for the incorporation of non-histone proteins, ensuring a smooth progression toward the final protamine-bound state.

Together, the coordinated activity of these chaperones is indispensable for managing the dynamic exchange of histone variants and orchestrating the epigenetic reprogramming required for functional sperm [81].

### Post-translational modifications

**Fig. 1:**
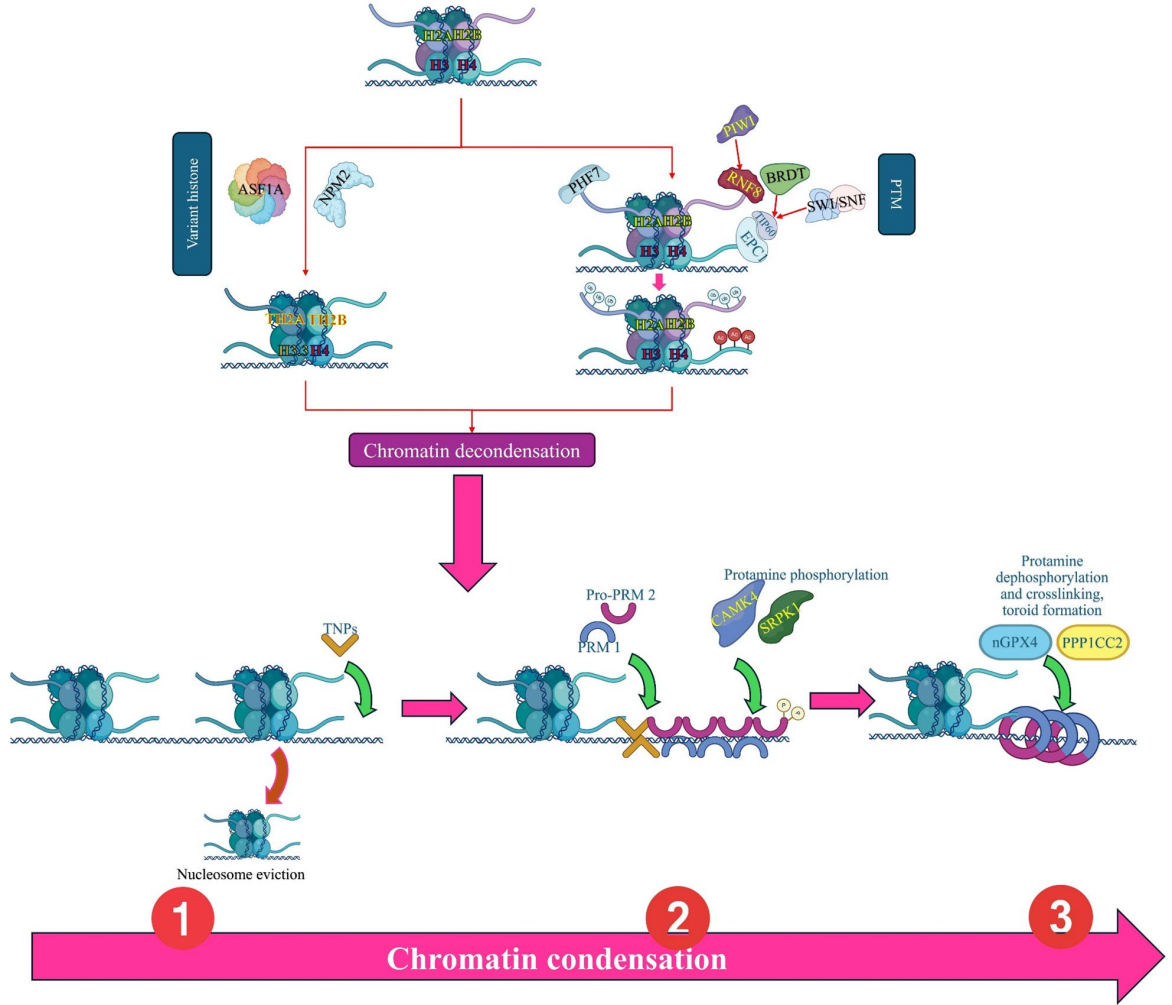
Molecular mechanisms of chromatin decondensation and histone-to-protamine replacement during spermatogenesis. The figure illustrates the molecular processes during spermatogenesis leading to chromatin decondensation and the replacement of histones with protamines. This pathway involves the substitution of canonical histones with histone variants (left) and post-translational modifications of histones (right), ultimately resulting in chromatin relaxation. Subsequently, transition nuclear proteins (TNPs) replace histones, followed by the incorporation of protamines into chromatin. Protamine phosphorylation, dephosphorylation, and crosslinking lead to the final condensation of chromatin into the toroidal structures characteristic of sperm nuclei. ***ASF1A***: Anti-Silencing Function 1 A, ***NPM2***: Nucleoplasmin 2, ***PTM***: Post-Translational Modification, ***RNF8***: Ring Finger Protein 8 (E3 ubiquitin-protein ligase), ***BRDT***: Bromodomain Testis-specific Protein, ***SWI/SNF***: Switch/Sucrose Non-Fermentable (chromatin remodeling complex), ***TNPs***: Transition Nuclear Proteins, ***Pro-PRM2***: Pro Protamine 2, ***PRM1***: Protamine 1, ***CAMK4***: Calcium/calmodulin-dependent protein kinase 4, ***SRPK1***: Serine/Arginine-Rich Protein-Specific Kinase 1, ***PPP1CC2***: Protein Phosphatase 1 Catalytic Subunit Gamma 2, ***nGPX4***: Nuclear Glutathione Peroxidase 4, ***EPC1***: Enhancer of Polycomb homolog 1, ***TIP60***: Tat-Interactive Protein, 60 kDa AND ***PIWI***: P-element Induced Wimpy Testis

PTMs constitute a central regulatory layer in spermiogenesis, orchestrating the adenosine triphosphate (ATP)-dependent transition from histones to protamines. These modifications fundamentally reshape nucleosome stability and histone–DNA interactions, thereby facilitating the large-scale chromatin remodeling required for sperm maturation [21, 63] (Fig. 1; Table 2). The process is tightly coordinated by Chromatin Remodeling Complexes (CRCs)—notably members of the SWI/SNF, ISWI, CHD, and INO80 families—that reposition or evict nucleosomes in an ATP-dependent manner The essential nature of CRCs is exemplified by BRG1, the catalytic ATPase of the SWI/SNF complex, whose deficiency results in incomplete histone removal and male infertility [25].

**Table 2:** Histone Post-Translational modifications in spermiogenesis

Modification type	Key Enzymes/Proteins	Histones/Residues Affected	Main functions	Phenotypic Consequences of Disruption	References
Acetylation	EPC1, TIP60 (NuA4 complex), SIRT1, BRDT, SMARCE1, PA200	H2A, H2B, H3, H4; H4K5, H4K8, H4K12, H4K16; testis-specific histone variants	Facilitates histone eviction, nucleosome destabilization, chromatin decompaction, recruitment of remodelers	Reduced fertility, abnormal sperm morphology, TNP2 mislocalization, defective histone replacement	[21, 58], 82– [94], 140– [142]
Ubiquitination	RNF8, MIWI (regulated by APC/C), L3MBTL2, PHF7	H2A, H2B; H4K16 (indirect)	Promotes histone removal, recruits MOF for H4K16ac, coordinates with methylation	Infertility, histone retention, defective protamine loading	[95–105]
Methylation	PYGO2, PHF7, SETD2, JHDM2A	H3K4me2/3 (active), H3K9me2/3, H3K27me3 (repressive), H3K79me2/3, H3K36me3	Balances transcription and compaction, activates Tnp/Prm genes, links to acetylation and ubiquitination	Disrupted chromatin condensation, infertility	[58], 106– [110, 143]
Phosphorylation	TSSK6, γH2AX, H4S1ph kinases	H2AX (Ser139), H4 (Ser1), H1T, HILS1, TH2A, TH2B	Meiotic recombination, sex chromosome inactivation, chromatin condensation	Infertility, impaired sperm morphology and motility, defective PRM2 processing	[43, 111–113]
Other Modifications	CDYL, PARP1, PARP2	Various histones; lysine crotonylation, butyrylation, malonylation, propionylation, succinylation	Marks active transcription sites, regulates histone replacement	Reduced fertility, histone retention, abnormal TNP1/PRM2 localization	[87, 114–116]

#### Acetylation

Histone hyperacetylation represents one of the earliest and most decisive epigenetic cues for histone eviction during spermiogenesis [21]. Sequential acetylation of histone H4 at lysine residues K5, K8, K12, and K16 (H4K5ac, H4K8ac, H4K12ac, and H4K16ac) follows a stage-specific pattern, with H4K16ac being enriched during the elongating spermatid phase [82]. These modifications directly reduce nucleosome–DNA affinity, promoting a more open and unstable chromatin structure [58, 83, 84]. The Nucleosome Acetyltransferase of H4 (NuA4) complex, composed of Enhancer of Polycomb homolog 1 (EPC1) and Tat-Interactive Protein 60 kDa (TIP60), catalyzes these acetylation events [58, 85]. The NAD⁺-dependent deacetylase Sirtuin 1 (SIRT1) also contributes to chromatin remodeling through dynamic regulation of histone acetylation [86, 87]. The testis-specific bromodomain protein BRDT recognizes acetylated H4 tails to drive chromatin reorganization [58, 88, 89] and interacts with the SWI/SNF subunit SMARCE1; disruption of BRDT function leads to male infertility [90, 91]. Final clearance of acetylated histones is mediated by the proteasome activator PA200, which facilitates ubiquitin-independent proteolysis. Deficiency of PA200 causes persistent retention of histones, including H2B, H3, and H4K16ac, reflecting impaired histone clearance during spermatid elongation [92–94].

#### Ubiquitination

Histone ubiquitination, particularly of H2A and H2B, is indispensable for histone degradation and turnover during spermiogenesis [95–97]. The E3 ubiquitin ligase Ring Finger Protein 8 (RNF8) plays a pivotal role in promoting histone removal [98, 99] in part by recruiting the histone acetyltransferase MOF (Males Absent on the First), which catalyzes H4K16 acetylation [100, 101]. RNF8 activity is regulated through APC/C (Anaphase-Promoting Complex/Cyclosome)-mediated degradation of P-element Induced Wimpy Testis) PIWI(proteins and interaction with Lethal 3 Malignant Brain Tumor-Like 2 (L3MBTL2 [102, 103]. In addition, the testis-specific E3 ligase PHD Finger Protein 7 (PHF7) functions as a molecular integrator that recognizes H3K4me2/3 via its PHD domain and mediates H2A ubiquitination [104]. Genetic ablation of Phf7 results in complete male infertility, underscoring its functional necessity [105].

#### Methylation

Histone methylation provides a dynamic platform that balances transcriptional activity and repression during spermiogenesis. Active (H3K4me2/3) and repressive (H3K9me2/3, H3K27me3) methylation marks often coexist, maintaining a poised chromatin state amenable to transition [106, 107]. The reader protein Pygopus family PHD finger 2 (PYGO2) bridges H3K4me3 recognition with histone acetyltransferase (HAT) activity, thereby linking methylation to acetylation-based chromatin remodeling [58, 108]. Activation of *Tnp* and *Prm* genes requires the methyltransferase SET domain-containing 2 (SETD2), the writer of H3K36me3 [104]. Conversely, the demethylase Jumonji domain-containing histone demethylase 2 A (JHDM2A), which removes H3K9me1/2, facilitates chromatin condensation and spermatid maturation [109, 110].

#### Phosphorylation

Phosphorylation acts as another key regulator of chromatin dynamics in elongating spermatids [111]. The phosphorylated histone variant γH2AX (phosphorylated H2AX) is abundant during this stage and is catalyzed by Testis-Specific Serine Kinase 6 (TSSK6). Deletion of Tssk6 leads to defective PRM2 processing and impaired chromatin compaction [112]. Moreover, phosphorylation of histone H4 at serine 1 (H4S1ph) contributes to chromatin accessibility and nucleoprotein exchange [113].

#### Other modifications

Beyond classical PTMs, emerging modifications add further layers of complexity to spermatid chromatin regulation [114, 115]. Lysine crotonylation (Kcr) has been identified as a hallmark of transcriptionally active regions and is modulated by Chromodomain Y like (CDYL). Loss of Cdyl disrupts the proper localization of TNP1 and PRM2, leading to abnormal chromatin condensation [87]. Additionally, Poly-ADP-ribosylation (PARsylation), catalyzed by Poly ADP-ribose polymerases 1 and 2, is indispensable for histone eviction; its disruption results in persistent retention of testis-specific histones H1T and HILS1 [116].

## Transition proteins 1 and 2: structure, expression, and functional roles in spermatid chromatin remodeling

Upon the loosening of chromatin facilitated by histone variants and their associated post-translational modifications, TNP1 and TNP2 play a crucial role in the subsequent stages of sperm chromatin packaging [31, 60]. These proteins are abundantly expressed in elongating spermatids, with *TNP1* expression being approximately 2.5 times higher than that of *TNP2* [117]. While TNP1 exhibits a relatively high degree of conservation across species, the sequence of *TNP2* shows greater variability, with its expression levels and overall abundance differing between various species [118]. The critical importance of both TNP1 and TNP2 in male fertility is well-established. For instance, studies involving the deletion of the TNP1 gene in mice have demonstrated severe consequences, including male infertility, the production of abnormally shaped sperm (particularly affecting head morphology), and a significant reduction in progressive sperm motility [119]. Furthermore, detailed analysis of sperm chromatin from these TNP1-deficient mice has revealed discernible changes in both the protein composition and the overall chromatin compaction levels, underscoring TNP1’s vital structural and functional contributions [120].

Traditionally, the prevailing understanding of transition proteins posited that they would temporarily occupy chromatin, acting as intermediate structures following the complete removal of histones, before the final incorporation of protamines [121]. In this model, TNPs were believed to transiently occupy the majority of the genome within elongating spermatids. However, more recent and in-depth investigations have challenged this strictly sequential view [122]. These studies suggest that the expression of *TNP*s does not necessarily precede that of protamines; instead, they are frequently co-expressed with protamines and other histone variants within spermatids. Direct observations in the spermatid nucleus at specific stages of spermatogenesis have confirmed their simultaneous presence [82]. Furthermore, a growing body of molecular, genetic, and biochemical data indicates that TNPs may not fully replace histones as a distinct, isolated step. Rather, it appears that different classes of nuclear proteins, encompassing histone variants, transition proteins, and protamines, function cooperatively and synchronously to facilitate a more direct transition from a histone-bound state to a protamine-bound state [64].

Despite their collaborative roles, TNP1 and TNP2 possess distinct functional properties. TNP1 exhibits a significantly higher affinity (more than 8 times greater) for single-stranded DNA (ssDNA) than for double-stranded DNA (dsDNA) [123]. When associated with DNA, TNP1 tends to form less stable structures and appears to play a role in facilitating DNA unwinding and relaxation within the nucleosomal structure. It is thought to achieve this by inserting itself between nucleic acid bases, which consequently reduces the DNA’s melting temperature, thus promoting a more open conformation [123]. In stark contrast, TNP2 demonstrates a much higher affinity (approximately 40 times greater) for dsDNA. This strong affinity allows TNP2 to effectively stabilize and condense DNA fibers across a wide range of ionic strengths [124]. These contrasting biochemical characteristics suggest that TNP2 likely performs a more direct and physical role in the histone replacement process and in the initial stages of chromatin compaction. The function of TNPs is also intricately associated with Topoisomerase II, an enzyme crucial for creating temporary breaks in the DNA molecule to relieve supercoiling tension, a process essential for the extensive chromatin remodeling occurring during spermatogenesis [123, 124].

## Final replacement with protamines

Protamines represent the final class of proteins to enter sperm chromatin, and they are exclusively responsible for the ultimate and exceptionally high compaction of paternal DNA. These small proteins, specifically expressed in sperm, are notably rich in the highly positive amino acid arginine, which is crucial for compacting paternal DNA during spermiogenesis. This extreme compaction allows the sperm head to adopt a highly condensed and hydrodynamic shape, a characteristic essential for efficient sperm motility and for protecting the paternal DNA during its transfer to the oocyte [31, 146]. From an evolutionary and biophysical standpoint, this hydrodynamic shape is a non-negotiable requirement for male fertility. Fertilization is, at its core, a competitive process, and sperm are the only human cells that must navigate a vast distance through an external environment. A nucleus with the volume, mass, and shape of a somatic cell would create immense hydrodynamic drag, rendering efficient motility impossible. Therefore, the evolution of protamine-based compaction is a direct adaptation for mobility, allowing the formation of the small, streamlined head required to win the “race” to the oocyte [125].

PRM1 typically consists of 49 or 50 amino acids in most mammals. It is characterized by three distinct domains: a central arginine-rich domain that primarily binds to the minor groove of DNA, and two terminal domains (N- and C-terminal) that contain cysteine residues. These cysteine residues are vital as they contribute to the formation of disulfide bonds between individual protamine molecules, which further stabilizes the highly condensed chromatin structure. Unlike other protamines, PRM1 is synthesized as a mature, complete protein and directly binds to chromatin without requiring further processing [126].

PRM2, in contrast to PRM1, has various isoforms and is expressed in certain species, including mice, humans, and monkeys. A key distinction is that PRM2 is initially synthesized as a longer precursor, referred to as precursor PRM2 (P-pro2). After this precursor enters the nucleus and binds to DNA, it undergoes a series of selective proteolytic processing steps, involving enzymatic cleavages, to remove its extra parts and yield its mature, functional form (PRM2). This proteolytic processing is an essential step for the proper function and efficacy of mature PRM2 [127, 128].

The mechanism by which protamines bind to DNA and facilitate compaction is fundamentally driven by charge neutralization. The highly positive charges of the arginine residues within protamines effectively neutralize the negative charges of the DNA phosphates. This neutralization allows DNA strands to come much closer together, leading to the formation of highly compacted structures known as toroids. Initially, both PRM1 and PRM2 undergo phosphorylation, a modification mediated by the enzymes Calcium/calmodulin-dependent protein kinase 4 (CAMK4) and Serine/Arginine-Rich Protein-Specific Kinase 1 (SRPK1). This initial phosphorylation step is absolutely essential for protamine binding to DNA. As mentioned, PRM2 first enters as a precursor and is subsequently processed into its final, functional form through proteolysis. Once bound, DNA wraps tightly around the protamines, forming the characteristic toroidal structures that result in extreme chromatin condensation [123, 129].

Following their binding to DNA, protamines must be dephosphorylated to ensure the stable association between the protamines and the DNA. This crucial dephosphorylation is carried out by the enzyme Protein Phosphatase 1 Catalytic Subunit Gamma 2 (PPP1CC2). PPP1CC2 is released from the Heat Shock Protein 70 chaperone complex with the assistance of the Heat shock 70 kDa protein 4 L co-chaperone, and it is then transported into the nucleus to perform its function. Specifically, the dephosphorylation of serine 56 in PRM2 is critical for the normal function of mature sperm. If a phosphate group remains at this particular position, it significantly impairs sperm maturation, highlighting the importance of this dephosphorylation step [123, 128].

The final stage of chromatin compaction and stabilization occurs during the sperm’s passage through the epididymis. At this stage, crucial structural reinforcements take place: disulfide bonds form between protamine molecules, particularly involving PRM2, and zinc ions (Zn^2+^) mediate additional cross-linking between PRM2 molecules. The enzyme nGPx4 (nuclear isoform of glutathione peroxidase 4) also plays a role in the formation of these disulfide bonds, ultimately bringing the final chromatin structure to an almost crystalline state. At the culmination of this intricate process, transcription is completely halted, and the sperm chromatin becomes extraordinarily condensed and transcriptionally inaccessible. This highly condensed state not only allows for minimal space to package the genetic information but also provides robust protection for the paternal genome from various physical, chemical, and enzymatic damages until the critical moment of fertilization [123, 130].

## Sperm chromatin organization

Sperm chromatin, compacted by protamines, exhibits specific organizational structures. These include Matrix Attachment Regions (MARs), which are DNA segments that link protamine-DNA toroids to the sperm’s nuclear matrix or scaffold. Their crucial role is to aid in the spatial organization and immobilization of DNA within the highly condensed structure of the sperm head. Additionally, there are Linker Regions (Inter-toroidal Regions). These DNA segments are situated between the toroidal units of compacted DNA. They are notably more susceptible to DNase (an enzyme that degrades DNA), meaning they are easily broken down when exposed to this enzyme. This sensitivity indicates a greater accessibility of these regions and suggests they likely play a significant role in regulating DNA access or preparing chromatin for its crucial reconstruction after fertilization [39].

## Remarkable reconstruction of sperm chromatin after fertilization: preparing for a new life

Upon the successful entry of sperm into the oocyte cytoplasm, the paternal chromatin, which has been highly compacted by protamines, undergoes a profound and multi-staged reconstruction. This dramatic chromatin reorganization within the sperm during fertilization is a critical initial process in sexual reproduction. It is absolutely essential for the proper formation of the paternal pronucleus – the initial nucleus derived from the sperm – and, ultimately, for the paternal genome’s vital contribution to embryonic development. This intricate and precisely coordinated process enables the activation of the paternal genome [24].

The first crucial step in this reconstruction, following sperm entry into the oocyte, involves the removal of protamines and the decondensation (unpacking) of sperm chromatin. This begins with the breaking of the disulfide bonds that stabilize the DNA-protamine complex, a feat achieved by the high concentration of the reducing molecule glutathione present in the maternal oocyte cytoplasm. The disruption of these bonds leads to the initial loosening of the chromatin structure [131]. Subsequently, the protamine proteins are gradually separated from the DNA and are systematically replaced by histones, which are abundantly supplied by the oocyte (maternal histones). This complex replacement process is facilitated by maternal nuclear chaperones, such as NPM2, and potentially other factors specifically involved in protamine removal. The immediate result of this stage is a significant decondensation of the paternal chromatin, effectively preparing it for the subsequent, more detailed stages of remodeling [132].

Following this initial unpacking, one of the most crucial events is the epigenetic reprogramming, characterized by extensive DNA demethylation of the paternal genome. DNA methylation, involving the addition of a methyl group to cytosines, is a fundamental epigenetic mark central to gene regulation. Upon the paternal nucleus’s entry into the oocyte cytoplasm, enzymes like Ten-Eleven Translocation 3 (TET3), and possibly other pathways involved in methylated cytosine oxidation, rapidly and significantly reduce the methylation patterns across paternal DNA. This massive wave of demethylation efficiently erases most methyl marks from the paternal genome, thereby enabling crucial gene re-activation and restoring totipotency – the ability to differentiate into any cell type – in the early embryo [132]. It is vital to note, however, that some specific paternal methylation sites, particularly those located in genomic imprinting regions (such as the imprinting control region or ICR in the H19 gene), are selectively protected from this widespread demethylation wave and remain untouched. Importantly, the selective preservation of DNA methylation at imprinted loci is not mediated solely by DNMT3A/L; it relies on the coordinated action of several complementary factors. While DNMT3A and DNMT3L are essential for the establishment of de novo DNA methylation marks in the male germline, including those at imprinted control regions (ICRs), they are not required for the maintenance of methylation at ICRs during the global demethylation phase after fertilization. DNMT1, in coordination with UHRF1, preserves these methylation patterns during DNA replication by recognizing hemi-methylated DNA and copying parental strand methylation onto the newly synthesized daughter strand, ensuring faithful epigenetic inheritance. PGC7 (Stella) binds to modified histones such as H3K9me2/3 and protects DNA methylation from active demethylation mediated by TET enzymes, preventing conversion of 5-methylcytosine to 5-hydroxymethylcytosine and subsequent loss. ZFP57, a KRAB zinc finger protein, recognizes methylated TGCCGC motifs at ICRs and recruits the cofactor TRIM28/KAP1, which stabilizes DNA methylation and promotes the formation of a local repressive chromatin environment. This complex further facilitates the recruitment of DNMTs and histone-modifying enzymes, reinforcing methylation and heterochromatin marks. Collectively, these factors act sequentially and cooperatively to ensure accurate deposition, maintenance, protection, and stabilization of DNA methylation in early embryogenesis [132–135]. Concurrently with the ongoing protamine removal, the third key stage involves the replacement with new histones and maternal histone variants. Histones and specific histone variants (such as H3.3), supplied from the oocyte’s cytoplasmic reserves, are loaded onto the paternal DNA. This crucial step meticulously re-establishes the fundamental nucleosomal structure. A dedicated set of maternal histone chaperones, notably including Histone Cell Cycle Regulator (HIRA), plays a pivotal role in this precise process of histone deposition onto paternal DNA, ensuring that the paternal DNA acquires a proper and fully functional nucleosomal architecture [132].

Immediately following histone incorporation, the fourth stage focuses on the regulation of epigenetic modifications on these newly deposited histones. New epigenetic modifications are actively established on these histones. These include both methylation and acetylation at specific lysine residues: particularly H3K27ac (lysine 27 acetylation), H3.3K4me (lysine 4 methylation), and H3.3K36me (lysine 36 methylation). All of these marks are strongly associated with active chromatin and signal a readiness for transcription. Importantly, H3.3K27 methylation is also concurrently observed in some closed and repetitive regions, where it paradoxically exerts a gene silencing role, demonstrating the nuanced control of chromatin states [132].

Finally, the culmination of these remodeling events leads to the paternal genome activation (Zygotic Genome Activation - ZGA) and the subsequent formation and fusion of the paternal pronucleus with the maternal pronucleus. Once the paternal chromatin has been sufficiently remodeled and meticulously prepared, the initial transcription of some paternal genes commences. This early transcriptional activity is known as Minor Zygotic Genome Activation (Minor ZGA) and often involves the expression of some non-coding RNAs and specific transposable elements, such as Murine Endogenous Retrovirus–Like)MERVL(. During this critical preparatory stage, the cellular system begins expressing a select portion of early genes in anticipation of the more extensive activation to follow. Subsequently, as the zygote undergoes its first few cell divisions, the Major Zygotic Genome Activation (Major ZGA) occurs. In this profound phase, the vast majority of embryonic genes, originating from both paternal and maternal genomes, begin to express, actively producing the proteins necessary for continued differentiation and normal embryonic growth. Concurrently with these genomic activations, the paternal pronucleus (containing the completely remodeled sperm chromatin) and the maternal pronucleus (containing the oocyte chromatin) meticulously migrate toward the center of the cell and eventually fuse. This ultimate fusion forms the diploid zygote nucleus, marking the true beginning of a new life [132].

## Limitations and precise regulations of chromatin reconstruction process

The process of sperm chromatin reconstruction after entering the oocyte is a complex, stepwise, and highly regulated biological pathway. While highly efficient biologically, this process has limitations in its functional capacity and requires precise temporal and molecular coordination.

### Limited capacity of oocyte cytoplasm

Experimental evidence indicates that the oocyte can fully reconstruct only a limited number of sperm nuclei. In studies where multiple sperm enter a single oocyte (polyspermy), it has been observed that if more than three sperm enter, only a limited number (usually up to three) can complete the full reconstruction stages and achieve mature pronucleus formation. Additional sperm often undergo only initial decondensation but fail in subsequent stages like recondensation, histone assembly, or pronucleus formation. This suggests a quantitative limitation in the reconstruction factors within the oocyte cytoplasm, likely including chaperone proteins, histone reserves, or phosphorylation enzymes [136].

### Energy-dependent and ATP-dependent process

Chromatin reconstruction is an energy-dependent process. Studies have shown that the assembly of maternal histones onto paternal DNA requires energy from ATP hydrolysis. When ATP production in the oocyte is inhibited by agents like antimycin A, paternal chromatin cannot proceed through the reconstruction stages: although decondensation may occur, it fails in recondensation, histone assembly, or pronuclear structure formation. This finding indicates that a significant portion of reconstruction, including protamine-histone replacement, is controlled by active, ATP-dependent pathways [137].

### Limited time window for reconstruction activity

The oocyte’s ability to reconstruct chromatin is restricted to a specific time frame, dependent on the meiotic maturation stage of the oocyte. The highest reconstruction activity is observed when the oocyte is transitioning from metaphase II. After oocyte activation and progression to interphase stages of the cell cycle, this activity significantly decreases or ceases entirely. This suggests that key reconstruction factors are either active only at this stage or are rapidly degraded or inactivated after this stage [138].

### Cell cycle regulation and coordination with chromatin reconstruction stages

The final stages of chromatin reconstruction—including complete decondensation of paternal chromatin within the pronucleus, nuclear protein entry, and reorganization of chromatin structure into a functional state—require a precise transition in the oocyte cell cycle from metaphase II to interphase. This transition is initiated by repeated fluctuations in calcium ion (Ca²⁺) concentration in the oocyte cytoplasm. The source of these fluctuations is a sperm-derived enzyme called phospholipase C-zeta (PLCζ), which is released upon sperm entry into the oocyte. This enzyme triggers IP₃ production in the oocyte, leading to calcium release from the endoplasmic reticulum. Calcium oscillations activate pathways that lead to cyclin B degradation. Cyclin B is the primary regulator of cyclin-dependent kinase 1 (CDK1) activity. In metaphase II, high levels of cyclin B maintain the oocyte in a blocked state. After cyclin B degradation, CDK1 activity rapidly decreases, and the oocyte exits M phase and enters interphase. This phase change is essential for the entry of nuclear proteins, reconstruction of pronuclear chromatin structure, and preparation for the zygote’s first mitotic division. Without this change, even if initial chromatin decondensation occurs, the reconstruction process will not be complete [139].

## Future directions and research opportunities

### Coordination of histone variants, transition proteins, and protamines

The precise spatial and temporal coordination among testis-specific histone variants, transition proteins, and protamines remains incompletely defined. Advanced single-cell proteomics and super-resolution imaging could help elucidate these dynamics.

### Functional role of post-translational modifications

Although numerous histone post-translational modifications have been identified during spermiogenesis, their functional interplay with the chromatin remodeling machinery is not yet fully understood. Integrative multi-omics approaches (ChIP-seq, mass spectrometry, transcriptomics) could clarify these regulatory networks.

### Mechanisms of selective histone retention

The molecular mechanisms that govern the selective retention of specific histones in mature sperm—and the functional significance of these nucleosomes for early embryonic gene regulation—remain to be elucidated.

### Post-fertilization chromatin reprogramming

The factors responsible for protamine removal and histone loading after fertilization, beyond known chaperones such as NPM2 and HIRA, are still not completely characterized.

### Paternal epigenetic inheritance

A deeper understanding of how environmental exposures in the male germline influence histone modifications, protamine composition, and DNA methylation is needed to reveal the mechanisms of paternal epigenetic inheritance and intergenerational effects.

### Integrative and interdisciplinary approaches

Addressing these unresolved questions will require collaborative efforts combining molecular biology, reproductive physiology, and computational modeling to build a comprehensive map of paternal genome packaging and its functional consequences for embryonic development.

## Conclusion

**Fig. 2:**
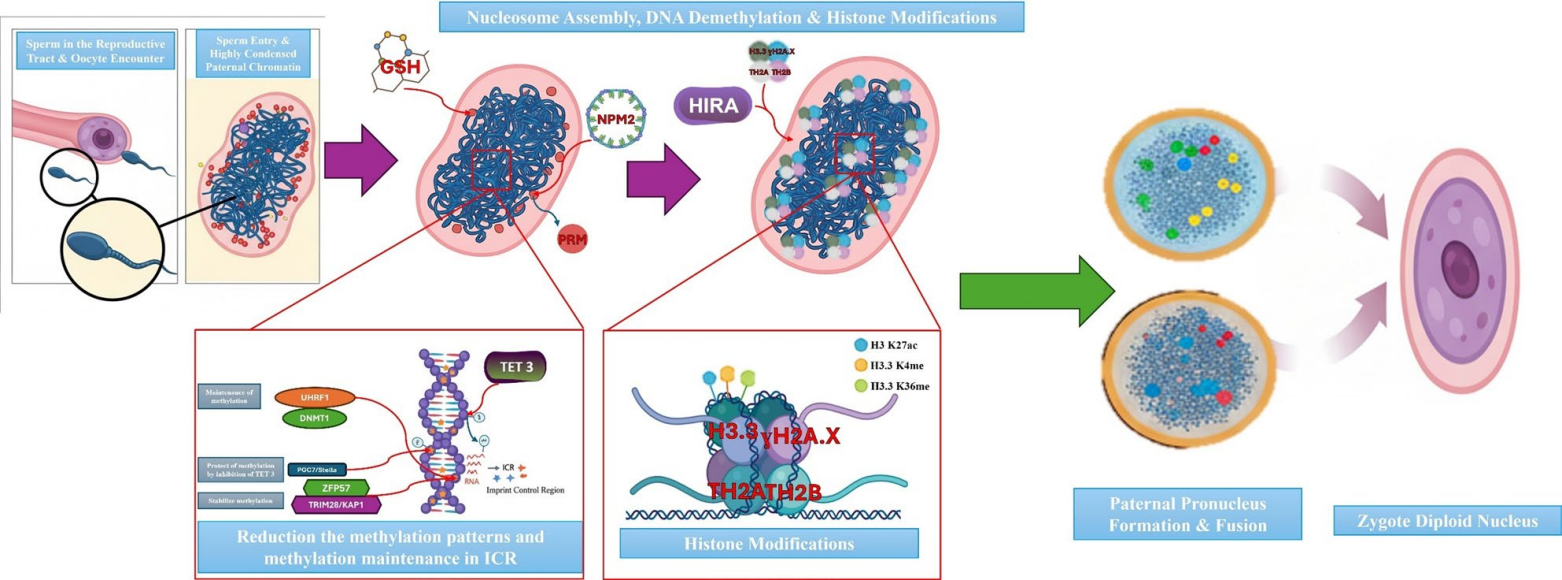
Epigenetic Reprogramming of the Paternal Genome after Fertilization. The schematic illustration depicts the sequential molecular events during paternal genome reprogramming following sperm–oocyte fusion. After sperm entry into the oocyte, the highly condensed paternal chromatin undergoes protamine removal mediated by GSH and NPM2, allowing chromatin relaxation. Maternal histone chaperone HIRA facilitates the replacement of protamines with histone variants (H3.3, H2A.X, TH2A, and TH2B), leading to nucleosome reassembly. Subsequently, active DNA demethylation occurs, primarily driven by ten–eleven translocation 3 (TET3), whereas maintenance and partial protection of ICRs involve DNMT1, TRIM28, and ZFP57. These processes are accompanied by dynamic histone post-translational modifications, including acetylation (H3K27ac) and methylation (H3K4me, H3K36me). The coordinated reorganization of the paternal chromatin culminates in the formation of the paternal pronucleus, which subsequently fuses with the maternal pronucleus to generate the diploid zygotic nucleus—marking the beginning of a new life. ***GSH***: Glutathione, ***NPM2***: Nucleoplasmin 2, ***PRM***: Protamine, ***HIRA***: Histone Cell Cycle Regulator, ***TET3***: Ten–Eleven Translocation 3, ***DNMT1***: DNA Methyltransferase 1, ***TRIM28***: Tripartite Motif-Containing Protein 28, ***ZFP57***: Zinc Finger Protein 57, ***ICR***: Imprinting Control Region, ***H3K27ac***: Histone H3 Lysine 27 Acetylation, ***H3K4me***: Histone H3 Lysine 4 Methylation, ***H3K36me***: Histone H3 Lysine 36 Methylation, ***H2A.X***,*** TH2A***,*** TH2B***: Histone Variants Involved in Chromatin Remodeling

In summary, the process of chromatin remodeling during spermatogenesis and its reversal after fertilization exemplifies a finely tuned regulatory system essential for male fertility and early embryonic development. Through the orchestrated action of histone variants, transition proteins, protamines, and their associated chaperones and modifications, the paternal genome achieves the structural and epigenetic state required for successful fertilization. Following fertilization, maternal factors rapidly reprogram this compacted genome to initiate zygotic transcription and embryogenesis. These highly coordinated events underscore the delicate balance between chromatin condensation and accessibility, and disruptions at any level can lead to infertility or developmental failure. A deeper mechanistic understanding of these processes will be crucial for advancing diagnostic and therapeutic approaches in reproductive medicine (Fig. 2).

## Data Availability

Data sharing is not applicable as no new data were generated or analyzed during this study.

## References

[1] Gaspa-Toneu L, Peters AH. Nucleosomes in mammalian sperm: conveying paternal epigenetic inheritance or subject to reprogramming between generations? Curr Opin Genet Dev. 2023. 10.1016/j.gde.2023.102034. 36893482 10.1016/j.gde.2023.102034 PMC10109108

[2] Minami K, Semeigazin A, Nakazato K, Maeshima K. Euchromatin and heterochromatin: implications for DNA accessibility and transcription. J Mol Biol. 2025. 10.1016/j.jmb.2025.169270 40482957

[3] Bar-Ziv R, Voichek Y, Barkai N. Chromatin dynamics during DNA replication. Genome Res. 2016;26:1245–56. 27225843 10.1101/gr.201244.115 PMC5052047

[4] Chu S, Li XH, Letcher RJ. Covalent adduct formation of histone with organophosphorus pesticides in vitro. Chem Biol Interact. 2024. 10.1016/j.cbi.2024.111095. 38844256 10.1016/j.cbi.2024.111095

[5] Bannister AJ, Kouzarides T. Regulation of chromatin by histone modifications. Cell Res. 2011;21:381–95. 21321607 10.1038/cr.2011.22 PMC3193420

[6] Morgunova E, Taipale J. Structural insights into the interaction between transcription factors and the nucleosome. Curr Opin Struct Biol. 2021;71:171–9. 34364091 10.1016/j.sbi.2021.06.016

[7] Bracci AN, Dallmann A, Ding Q, Hubisz MJ, Caballero M, Koren A. The evolution of the human DNA replication timing program. Proc Natl Acad Sci U S A. 2023. 10.1073/pnas.2213896120. 36848554 10.1073/pnas.2213896120 PMC10013799

[8] Karagyozova T, Almouzni G. Replicating chromatin in the nucleus: a histone variant perspective. Curr Opin Cell Biol. 2024. 10.1016/j.ceb.2024.102397. 38981199 10.1016/j.ceb.2024.102397

[9] Serdyuk A, Allers T. DNA replication in time and space: the archaeal dimension. Dna. 2025;5:24.

[10] Tang X, Yao Y, Li G, Gan H. Insights into the synchronization between DNA replication and parental histone recycling. Biochem Soc Trans. 2025. 10.1042/BST20253014. 40380884 10.1042/BST20253014 PMC12224953

[11] Reverón-Gómez N, González-Aguilera C, Stewart-Morgan KR, Petryk N, Flury V, Graziano S, et al. Accurate recycling of parental histones reproduces the histone modification landscape during DNA replication. Mol Cell. 2018;72:239-249.e5. 30146316 10.1016/j.molcel.2018.08.010 PMC6202308

[12] Thiriet C. Usage of the H3 variants during the S-phase of the cell cycle in physarum polycephalum. Nucleic Acids Res. 2022;50:2536–48. 35137186 10.1093/nar/gkac060 PMC8934661

[13] Dreyer J, Ricci G, van den Berg J, Bhardwaj V, Funk J, Armstrong C, et al. Acute multi-level response to defective de novo chromatin assembly in S-phase. Mol Cell. 2024;84:4945. 39626659 10.1016/j.molcel.2024.11.036

[14] Vijayalakshmi P, Gowdham M, Dinesh DC, Sibiya A, Vaseeharan B, Selvaraj C. Unveiling the guardians of the genome: the dynamic role of histones in DNA organization and disease. Adv Protein Chem Struct Biol. 2025;143:39–68. 39843143 10.1016/bs.apcsb.2024.08.001

[15] Nadal S, Raj R, Mohammed S, Davis BG. Synthetic post-translational modification of histones. Curr Opin Chem Biol. 2018;45:35–47. 29501025 10.1016/j.cbpa.2018.02.004

[16] Song HY, Shen R, Mahasin H, Guo YN, Wang DG. DNA replication: mechanisms and therapeutic interventions for diseases. MedComm. 2023. 10.1002/mco2.210. 36776764 10.1002/mco2.210 PMC9899494

[17] Wan H, Cao L, Wang P, Hu H, Guo R, Chen J, et al. Genome-wide mapping of main histone modifications and coordination regulation of metabolic genes under salt stress in pea (*Pisum sativum* L). Hortic Res. 2024. 10.1093/hr/uhae259. 39664693 10.1093/hr/uhae259 PMC11630261

[18] Igolkina AA, Zinkevich A, Karandasheva KO, Popov AA, Selifanova MV, Nikolaeva D, et al. H3K4me3, H3K9ac, H3K27ac, H3K27me3 and H3K9me3 histone tags suggest distinct regulatory evolution of open and condensed chromatin landmarks. Cells. 2019. 10.3390/cells8091034. 31491936 10.3390/cells8091034 PMC6770625

[19] Zhang T, Cooper S, Brockdorff N. The interplay of histone modifications – writers that read. EMBO Rep. 2015;16:1467–81. 26474904 10.15252/embr.201540945 PMC4641500

[20] Kovalev MA, Mamaeva NY, Kristovskiy NV, Feskin PG, Vinnikov RS, Oleinikov PD, et al. Epigenome engineering using dCas systems for biomedical applications and biotechnology: current achievements, opportunities and challenges. Int J Mol Sci. 2025. 10.3390/ijms26136371. 40650152 10.3390/ijms26136371 PMC12250444

[21] Bao J, Bedford MT. Epigenetic regulation of the histone-to-protamine transition during spermiogenesis. Reproduction. 2016;151:R55–70. 26850883 10.1530/REP-15-0562 PMC4896072

[22] Gómez-Giménez B, Lacalle E, Martínez-Pastor F, Soriano-Úbeda C. Analysis of the chromatin structure by chromomycin A3 (CMA3) and flow cytometry. Methods Mol Biol. 2025;2897:507–15. 40202656 10.1007/978-1-0716-4406-5_33

[23] Oliva R, Dixon GH. Vertebrate protamine genes and the histone-to-protamine replacement reaction. Prog Nucleic Acid Res Mol Biol. 1991;40:25–94. 2031084 10.1016/s0079-6603(08)60839-9

[24] Gou L-T, Lim D-H, Ma W, Aubol BE, Hao Y, Wang X, et al. Initiation of parental genome reprogramming in fertilized oocyte by splicing kinase SRPK1-catalyzed protamine phosphorylation. Cell. 2020;180:1212-1227.e14. 10.1016/j.cell.2020.02.020. 32169215 10.1016/j.cell.2020.02.020 PMC7190278

[25] Cui Y, Deng J, Zhang Y, Du L, Jiang F, Li C, et al. Epigenetic regulation by DNA methylation, histone modifications and chromatin remodeling complexes in controlling spermatogenesis and their dysfunction with male infertility. Cell Mol Life Sci. 2025;82:343. 41051601 10.1007/s00018-025-05831-5 PMC12500515

[26] Rafa AY, Filliaux S, Lyubchenko YL. Nanoscale characterization of interaction of nucleosomes with H1 linker histone. Int J Mol Sci. 2025;26. 10.3390/ijms26010303 PMC11719560 39796159

[27] Bradbury EM. K. E. Van Holde. Chromatin. Series in molecular biology. Springer-Verlag, New York. 1988. 530 pp. $98.00. J Mol Recognit. 1989;2.

[28] Annunziato AT. DNA Packaging: Nucleosomes and Chromatin. Nat Educ. 2008; Available from: http://www

[29] Olins AL, Olins DE. Spheroid chromatin units (v bodies). Science (80-). 1974;183:330–2. 10.1126/science.183.4122.330 4128918

[30] Cho C, Willis WD, Goulding EH, Jung-Ha H, Choi YC, Hecht NB, et al. Haploinsufficiency of protamine-1 or -2 causes infertility in mice. Nat Genet. 2001;28:82–6. 11326282 10.1038/ng0501-82

[31] Moritz L, Hammoud SS. The art of packaging the sperm genome: molecular and structural basis of the histone-to-protamine exchange. Front Endocrinol (Lausanne). 2022. 10.3389/fendo.2022.895502. 35813619 10.3389/fendo.2022.895502 PMC9258737

[32] Brykczynska U, Hisano M, Erkek S, Ramos L, Oakeley EJ, Roloff TC, et al. Repressive and active histone methylation mark distinct promoters in human and mouse spermatozoa. Nat Struct Mol Biol. 2010;17:679–87. 20473313 10.1038/nsmb.1821

[33] Hammoud SS, Nix DA, Zhang H, Purwar J, Carrell DT, Cairns BR. Distinctive chromatin in human sperm packages genes for embryo development. Nature. 2009;460:473–8. 19525931 10.1038/nature08162 PMC2858064

[34] Jung YH, Sauria MEG, Lyu X, Cheema MS, Ausio J, Taylor J, et al. Chromatin states in mouse sperm correlate with embryonic and adult regulatory landscapes. Cell Rep. 2017;18:1366–82. 28178516 10.1016/j.celrep.2017.01.034 PMC5313040

[35] Erkek S, Hisano M, Liang CY, Gill M, Murr R, Dieker J, et al. Molecular determinants of nucleosome retention at CpG-rich sequences in mouse spermatozoa. Nat Struct Mol Biol. 2013;20:868–75. 23770822 10.1038/nsmb.2599

[36] Gold HB, Jung YH, Corces VG. Not just heads and tails: the complexity of the sperm epigenome. J Biol Chem. 2018;293:13815–20. 29507096 10.1074/jbc.R117.001561 PMC6130957

[37] Ribas-Maynou J, Nguyen H, Wu H, Ward WS. Functional aspects of sperm chromatin organization. Results Probl Cell Differ. 2022;70:295–311. 36348112 10.1007/978-3-031-06573-6_10 PMC9671218

[38] Allen MJ, Bradbury EM, Balhorn R. AFM analysis of DNA-protamine complexes bound to mica. Nucleic Acids Res. 1997;25:2221–6. 9153324 10.1093/nar/25.11.2221 PMC146714

[39] De Jonge CJ, Barratt CLR. The sperm cell: production, maturation, fertilization, regeneration. Sperm cell prod. Matur Fertil Regen. 2017.

[40] Wyrobek AJ, Meistrich ML, Furrer R, Bruce WR. Physical characteristics of mouse sperm nuclei. Biophys J. 1976;16:811–25. 938720 10.1016/S0006-3495(76)85730-X PMC1334902

[41] Ward WS. Organization of sperm DNA by the nuclear matrix. Am J Clin Exp Urol. 2018;6:87–92. 29666836 PMC5902726

[42] Castillo J, Gay M, De La Iglesia A, Arauz-Garofalo G, Vilanova M, Leiva M, et al. Alterations in the abundance of protamine proteoforms related to sperm chromatin packaging, obesity, and age in normozoospermic men. Mol Hum Reprod. 2025. 10.1093/molehr/gaaf019. 40411759 10.1093/molehr/gaaf019

[43] Torres-Flores U, Hernández-Hernández A. The interplay between replacement and retention of histones in the sperm genome. Front Genet. 2020. 10.3389/fgene.2020.00780. 32765595 10.3389/fgene.2020.00780 PMC7378789

[44] Talbert PB, Henikoff S. Histone variants ancient wrap artists of the epigenome. Nat Rev Mol Cell Biol. 2010;11:264–75. 20197778 10.1038/nrm2861

[45] Kowalski A, Pałyga J. Linker histone subtypes and their allelic variants. Cell Biol Int. 2012;36:981–96. 23075301 10.1042/CBI20120133

[46] McCarrey JR, Geyer CB, Yoshioka H. Epigenetic regulation of testis-specific gene expression. Ann N Y Acad Sci. 2005;1061:226–42. 16467272 10.1196/annals.1336.025

[47] Govin J, Escoffier E, Rousseaux S, Kuhn L, Ferro M, Thévenon J, et al. Pericentric heterochromatin reprogramming by new histone variants during mouse spermiogenesis. J Cell Biol. 2007;176:283–94. 17261847 10.1083/jcb.200604141 PMC2063955

[48] Fyodorov DV, Zhou BR, Skoultchi AI, Bai Y. Emerging roles of linker histones in regulating chromatin structure and function. Nat Rev Mol Cell Biol. 2018;19:192–206. 29018282 10.1038/nrm.2017.94 PMC5897046

[49] Medrzycki M, Zhang Y, Cao K, Fan Y. Expression analysis of mammalian Linker-histone subtypes. J Vis Exp. 2012. 10.3791/3577 PMC3415166 22453355

[50] Drabent B, Benavente R, Hoyer-Fender S. Histone H1t is not replaced by H1.1 or H1.2 in pachytene spermatocytes or spermatids of H1t-deficient mice. Cytogenet Genome Res. 2003;103:307–13. 15051953 10.1159/000076818

[51] Fantz DA, Hatfield WR, Horvath G, Kistler MK, Kistler WS. Mice with a targeted disruption of the H1t gene are fertile and undergo normal changes in structural chromosomal proteins during spermiogenesis. Biol Reprod. 2001;64:425–31. 11159343 10.1095/biolreprod64.2.425

[52] Pascal C, Zonszain J, Hameiri O, Gargi-Levi C, Lev-Maor G, Tammer L, et al. Human histone H1 variants impact splicing outcome by controlling RNA polymerase II elongation. Mol Cell. 2023;83:3801-3817.e8. 37922872 10.1016/j.molcel.2023.10.003

[53] Drabent B, Saftig P, Bode C, Doenecke D. Spermatogenesis proceeds normally in mice without linker histone H1t. Histochem Cell Biol. 2000;113:433–42. 10933220 10.1007/s004180000146

[54] Martianov I, Brancorsini S, Catena R, Gansmuller A, Kotaja N, Parvinen M, et al. Polar nuclear localization of H1T2, a histone H1 variant, required for spermatid elongation and DNA condensation during spermiogenesis. Proc Natl Acad Sci U S A. 2005;102:2808–13. 15710904 10.1073/pnas.0406060102 PMC549447

[55] Tanaka H, Iguchi N, Isotani A, Kitamura K, Toyama Y, Matsuoka Y, et al. HANP1/H1T2, a novel histone H1-like protein involved in nuclear formation and sperm fertility. Mol Cell Biol. 2005;25:7107–19. 16055721 10.1128/MCB.25.16.7107-7119.2005 PMC1190238

[56] Yan W, Ma L, Burns KH, Matzuk MM. HILS1 is a spermatid-specific linker histone H1-like protein implicated in chromatin remodeling during mammalian spermiogenesis. Proc Natl Acad Sci U S A. 2003;100:10546–51. 12920187 10.1073/pnas.1837812100 PMC193598

[57] Mishra LN, Shalini V, Gupta N, Ghosh K, Suthar N, Bhaduri U, et al. Spermatid-specific linker histone HILS1 is a poor condenser of DNA and chromatin and preferentially associates with LINE-1 elements. Epigenetics Chromatin. 2018. 10.1186/s13072-018-0214-0. 30068355 10.1186/s13072-018-0214-0 PMC6069787

[58] Wang T, Gao H, Li W, Liu C. Essential role of histone replacement and modifications in male fertility. Front Genet. 2019;10:962. 31649732 10.3389/fgene.2019.00962 PMC6792021

[59] Soboleva TA, Parker BJ, Nekrasov M, Hart-Smith G, Tay YJ, Tng WQ, et al. A new link between transcriptional initiation and pre-mRNA splicing: the RNA binding histone variant H2A.B. PLoS Genet. 2017. 10.1371/journal.pgen.1006633. 28234895 10.1371/journal.pgen.1006633 PMC5345878

[60] Firouzabadi AM, Henkel R, Tofighi Niaki M, Fesahat F. Adverse effects of nicotine on human sperm nuclear proteins. World J Mens Health. 2024. 10.5534/wjmh.240072. 39028130 10.5534/wjmh.240072 PMC11937351

[61] Padavattan S, Shinagawa T, Hasegawa K, Kumasaka T, Ishii S, Kumarevel T. Structural and functional analyses of nucleosome complexes with mouse histone variants TH2a and TH2b, involved in reprogramming. Biochem Biophys Res Commun. 2015;464:929–35. 26188507 10.1016/j.bbrc.2015.07.070

[62] Padavattan S, Thiruselvam V, Shinagawa T, Hasegawa K, Kumasaka T, Ishii S, et al. Structural analyses of the nucleosome complexes with human testis-specific histone variants, hTh2a and hTh2b. Biophys Chem. 2017;221:41–8. 27992841 10.1016/j.bpc.2016.11.013

[63] Shinagawa T, Huynh LM, Takagi T, Tsukamoto D, Tomaru C, Kwak HG, et al. Disruption of TH2a and TH2b genes causes defects in spermatogenesis. Development. 2015;142:1287–92. 25742800 10.1242/dev.121830

[64] Barral S, Morozumi Y, Tanaka H, Montellier E, Govin J, de Dieuleveult M, et al. Histone variant H2A.L.2 guides transition protein-dependent protamine assembly in male germ cells. Mol Cell. 2017;66:89-101.e8. 28366643 10.1016/j.molcel.2017.02.025

[65] Soboleva TA, Nekrasov M, Pahwa A, Williams R, Huttley GA, Tremethick DJ. A unique H2A histone variant occupies the transcriptional start site of active genes. Nat Struct Mol Biol. 2012;19:25–31. 10.1038/nsmb.2161 22139013

[66] Anuar ND, Kurscheid S, Field M, Zhang L, Rebar E, Gregory P, et al. Gene editing of the multi-copy H2A.B gene and its importance for fertility. Genome Biol. 2019. 10.1186/s13059-019-1633-3. 30704500 10.1186/s13059-019-1633-3 PMC6357441

[67] Singh I, Parte P. Heterogeneity in the epigenetic landscape of murine testis-specific histone variants TH2A and TH2B sharing the same bi-directional promoter. Front Cell Dev Biol. 2021. 10.3389/fcell.2021.755751. 34938732 10.3389/fcell.2021.755751 PMC8685415

[68] Montellier E, Boussouar F, Rousseaux S, Zhang K, Buchou T, Fenaille F, et al. Chromatin-to-nucleoprotamine transition is controlled by the histone H2B variant TH2B. Genes Dev. 2013;27:1680–92. 23884607 10.1101/gad.220095.113 PMC3744726

[69] Ding D, Pang MYH, Deng M, Nguyen TT, Liu Y, Sun X, et al. Testis-specific H2B.W1 disrupts nucleosome integrity by reducing DNA–histone interactions. Nucleic Acids Res. 2024;52:11612–25. 39329259 10.1093/nar/gkae825 PMC11514470

[70] Hoghoughi N, Barral S, Vargas A, Rousseaux S, Khochbin S. Histone variants: essential actors in male genome programming. J Biochem. 2018;163:97–103. 29165574 10.1093/jb/mvx079

[71] Singh I, Parte P. Genome-wide profiling of the epigenetic landscape of histone variant TH2B in murine oocytes and pre-implantation embryos. Reproduction. 2025;169. 10.1530/REP-24-0035 39447008

[72] Teimouri M, Najaran H, Hosseinzadeh A, Mazoochi T. Association between two common transitions of H2BFWT gene and male infertility: a case–control, meta, and structural analysis. Andrology. 2018;6:306–16. 29453813 10.1111/andr.12464

[73] Rafatmanesh A, Nikzad H, Ebrahimi A, Karimian M, Zamani T. Association of the c.-9C > T and c.368A > G transitions in H2BFWT gene with male infertility in an Iranian population. Andrologia. 2018. 10.1111/and.12805. 28370107 10.1111/and.12805

[74] Amor H, Juhasz-Böss I, Bibi R, Hammadeh ME, Jankowski PM. H2BFWT variations in sperm DNA and its correlation to pregnancy. Int J Mol Sci. 2024. 10.3390/ijms25116048. 38892236 10.3390/ijms25116048 PMC11172515

[75] Amatori S, Tavolaro S, Gambardella S, Fanelli M. The dark side of histones: genomic organization and role of oncohistones in cancer. Clin Epigenetics. 2021. 10.1186/s13148-021-01057-x. 33827674 10.1186/s13148-021-01057-x PMC8025322

[76] Tang MCW, Jacobs SA, Mattiske DM, Soh YM, Graham AN, Tran A, et al. Contribution of the two genes encoding histone variant H3.3 to viability and fertility in mice. PLoS Genet. 2015;11:1–23. 10.1371/journal.pgen.1004964 PMC4335506 25675407

[77] Tafessu A, O’Hara R, Martire S, Dube AL, Saha P, Gant VU, et al. H3.3 contributes to chromatin accessibility and transcription factor binding at promoter-proximal regulatory elements in embryonic stem cells. Genome Biol. 2023. 10.1186/s13059-023-02867-3. 36782260 10.1186/s13059-023-02867-3 PMC9926682

[78] Ueda J, Harada A, Urahama T, Machida S, Maehara K, Hada M, et al. Testis-specific histone variant H3t gene is essential for entry into spermatogenesis. Cell Rep. 2017;18:593–600. 28099840 10.1016/j.celrep.2016.12.065

[79] Shiraishi K, Shindo A, Harada A, Kurumizaka H, Kimura H, Ohkawa Y, et al. Roles of histone H3.5 in human spermatogenesis and spermatogenic disorders. Andrology. 2018;6:158–65. 29179259 10.1111/andr.12438

[80] Emelyanov AV, Barcenilla-Merino D, Loppin B, Fyodorov DV. APOLLO, a testis-specific *Drosophila* ortholog of importin-4, mediates the loading of protamine-like protein Mst77F into sperm chromatin. J Biol Chem. 2023. 10.1016/j.jbc.2023.105212. 37660905 10.1016/j.jbc.2023.105212 PMC10520872

[81] Rajam SM, Varghese PC, Dutta D. Histone chaperones as cardinal players in development. Front Cell Dev Biol. 2022. 10.3389/fcell.2022.767773. 35445016 10.3389/fcell.2022.767773 PMC9014011

[82] Ketchum CC, Larsen CD, McNeil A, Meyer-Ficca ML, Meyer RG. Early histone H4 acetylation during chromatin remodeling in equine spermatogenesis. Biol Reprod. 2018;98:115–29. 29186293 10.1093/biolre/iox159

[83] Zhang R, Erler J, Langowski J. Histone acetylation regulates chromatin accessibility: role of H4K16 in inter-nucleosome interaction. Biophys J. 2017;112:450–9. 27931745 10.1016/j.bpj.2016.11.015 PMC5300776

[84] Li R, Lin X. Connected chromatin amplifies acetylation-modulated nucleosome interactions. Biochemistry. 2025;64:1222–32. 40029962 10.1021/acs.biochem.4c00647 PMC11925056

[85] Dong Y, Isono K, Ohbo K, Endo TA, Ohara O, Maekawa M, et al. EPC1/TIP60-mediated histone acetylation facilitates spermiogenesis in mice. Mol Cell Biol. 2017;37:e00082-17. 10.1128/MCB.00082-17. 28694333 10.1128/MCB.00082-17 PMC5599718

[86] Bell EL, Nagamori I, Williams EO, Del Rosario AM, Bryson BD, Watson N, et al. SirT1 is required in the male germ cell for differentiation and fecundity in mice. Development. 2014;141:3495–504. 10.1242/dev.110627. 25142464 10.1242/dev.110627 PMC4197722

[87] Liu S, Yu H, Liu Y, Liu X, Zhang Y, Bu C, et al. Chromodomain protein CDYL acts as a crotonyl-CoA hydratase to regulate histone crotonylation and spermatogenesis. Mol Cell. 2017;67:853-866.e5. 28803779 10.1016/j.molcel.2017.07.011

[88] Manterola M, Brown TM, Oh MY, Garyn C, Gonzalez BJ, Wolgemuth DJ. BRDT is an essential epigenetic regulator for proper chromatin organization, silencing of sex chromosomes and crossover formation in male meiosis. PLoS Genet. 2018. 10.1371/journal.pgen.1007209. 29513658 10.1371/journal.pgen.1007209 PMC5841650

[89] Kim CR, Noda T, Kim H, Kim G, Park S, Na Y, et al. PHF7 modulates BRDT stability and histone-to-protamine exchange during spermiogenesis. Cell Rep. 2020. 10.1016/j.celrep.2020.107950. 32726616 10.1016/j.celrep.2020.107950

[90] Gaucher J, Boussouar F, Montellier E, Curtet S, Buchou T, Bertrand S, et al. Bromodomain-dependent stage-specific male genome programming by Brdt. EMBO J. 2012;31:3809–20. 22922464 10.1038/emboj.2012.233 PMC3463845

[91] Kashiwagi K, Yoshida J, Kimura H, Shinjo K, Kondo Y, Horie K. Mutation of the SWI/SNF complex component Smarce1 decreases nucleosome stability in embryonic stem cells and impairs differentiation. J Cell Sci. 2024. 10.1242/jcs.260467. 38357971 10.1242/jcs.260467

[92] Zhang ZH, Jiang TX, Chen L, Bin, Zhou W, Liu Y, Gao F et al. Proteasome subunit α4s is essential for formation of spermatoproteasomes and histone degradation during meiotic DNA repair in spermatocytes. J Biol Chem. 2021;296. 10.1074/jbc.RA120.016485 PMC7949063 33262216

[93] Xiong Y, Yu C, Zhang Q. Ubiquitin-proteasome system–regulated protein degradation in spermatogenesis. Cells. 2022;11. 10.3390/cells11061058 PMC8947704 35326509

[94] Qian MX, Pang Y, Liu CH, Haratake K, Du BY, Ji DY, et al. XAcetylation-mediated proteasomal degradation of core histones during DNA repair and spermatogenesis. Cell. 2013;153:1012. 23706739 10.1016/j.cell.2013.04.032 PMC3983474

[95] Deng L, Meng T, Chen L, Wei W, Wang P. The role of ubiquitination in tumorigenesis and targeted drug discovery. Signal Transduct Target Ther. 2020;5. 10.1038/s41392-020-0107-0 PMC7048745 32296023

[96] Ma T, Keller JA, Yu X. RNF8-dependent histone ubiquitination during DNA damage response and spermatogenesis. Acta Biochim Biophys Sin (Shanghai). 2011;43:339–45. 21444325 10.1093/abbs/gmr016 PMC3080603

[97] Adams SR, Maezawa S, Alavattam KG, Abe H, Sakashita A, Shroder M et al. RNF8 and SCML2 cooperate to regulate ubiquitination and H3K27 acetylation for escape gene activation on the sex chromosomes. PLoS Genet. 2018;14. 10.1371/journal.pgen.1007233 PMC5834201 29462142

[98] Guo Y, Song Y, Guo Z, Hu M, Liu B, Duan H, et al. Function of RAD6B and RNF8 in spermatogenesis. Cell Cycle. 2018;17:162–73. 28825854 10.1080/15384101.2017.1361066 PMC5884393

[99] Gou LT, Kang JY, Dai P, Wang X, Li F, Zhao S, et al. Ubiquitination-deficient mutations in human Piwi cause male infertility by impairing histone-to-protamine exchange during spermiogenesis. Cell. 2017;169:1090-1104.e13. 28552346 10.1016/j.cell.2017.04.034 PMC5985145

[100] González-Bermúdez L, Genescà A, Terradas M, Martín M. Role of H4K16 acetylation in 53BP1 recruitment to double-strand break sites in in vitro aged cells. Biogerontology. 2022;23:499–514. 35851632 10.1007/s10522-022-09979-6 PMC9388460

[101] Kumar R, Horikoshi N, Singh M, Gupta A, Misra HS, Albuquerque K et al. Chromatin modifications and the DNA damage response to ionizing radiation. Front Oncol. 2013;2. 10.3389/fonc.2012.00214 PMC3551241 23346550

[102] Abe H, Meduri R, Li Z, Andreassen PR, Namekawa SH. RNF8 is not required for histone-to-protamine exchange in spermiogenesis. Biol Reprod. 2021;105:1154–9. 34225362 10.1093/biolre/ioab132 PMC8599036

[103] Meng C, Liao J, Zhao D, Huang H, Qin J, Lee TL, et al. L3MBTL2 regulates chromatin remodeling during spermatogenesis. Cell Death Differ. 2019;26:2194–207. 30760872 10.1038/s41418-019-0283-z PMC6889272

[104] Wang X, Kang JY, Wei L, Yang X, Sun H, Yang S, et al. PHF7 is a novel histone H2A E3 ligase prior to histone-to-protamine exchange during spermiogenesis. Development. 2019. 10.1242/dev.175547. 31189663 10.1242/dev.175547

[105] Lee HS, Bang I, You J, Jeong TK, Kim CR, Hwang M, et al. Molecular basis for PHF7-mediated ubiquitination of histone H3. Genes Dev. 2023;37:984–97. 37993255 10.1101/gad.350989.123 PMC10760634

[106] Barbero G, de Sousa Serro MG, Perez Lujan C, Vitullo AD, González CR, González B. Transcriptome profiling of histone writers/erasers enzymes across spermatogenesis, mature sperm and pre-cleavage embryo: implications in paternal epigenome transitions and inheritance mechanisms. Front Cell Dev Biol. 2023;11. 10.3389/fcell.2023.1086573 PMC9911891 36776561

[107] Deb M, Kar S, Sengupta D, Shilpi A, Parbin S, Rath SK, et al. Chromatin dynamics: H3K4 methylation and H3 variant replacement during development and in cancer. Cell Mol Life Sci. 2014;71:3439–63. 24676717 10.1007/s00018-014-1605-4 PMC11113154

[108] Nair M, Nagamori I, Sun P, Mishra DP, Rhéaume C, Li B, et al. Nuclear regulator Pygo2 controls spermiogenesis and histone H3 acetylation. Dev Biol. 2008;320:446–55. 18614164 10.1016/j.ydbio.2008.05.553 PMC2553271

[109] Dimitrova E, Turberfield AH, Klose RJ. Histone demethylases in chromatin biology and beyond. EMBO Rep. 2015;16:1620–39. 26564907 10.15252/embr.201541113 PMC4687429

[110] Okada Y, Scott G, Ray MK, Mishina Y, Zhang Y. Histone demethylase JHDM2A is critical for Tnp1 and Prm1 transcription and spermatogenesis. Nature. 2007;450:119–23. 17943087 10.1038/nature06236

[111] Jiang H, Gao Q, Zheng W, Yin S, Wang L, Zhong L et al. MOF influences meiotic expansion of H2AX phosphorylation and spermatogenesis in mice. PLoS Genet. 2018;14. 10.1371/journal.pgen.1007300 PMC6019819 29795555

[112] Merighi A, Gionchiglia N, Granato A, Lossi L. The phosphorylated form of the histone h2ax (γh2ax) in the brain from embryonic life to old age. Molecules. 2021;26. 10.3390/molecules26237198 PMC8659122 34885784

[113] Zhang ZH, Mu SM, Guo MS, Wu JL, Li YQ, Zhang H, et al. Dynamics of histone H2A, H4 and HS1ph during spermatogenesis with a focus on chromatin condensation and maturity of spermatozoa. Sci Rep. 2016. 10.1038/srep25089. 27121047 10.1038/srep25089 PMC4848542

[114] Tan M, Luo H, Lee S, Jin F, Yang JS, Montellier E, et al. Identification of 67 histone marks and histone lysine crotonylation as a new type of histone modification. Cell. 2011;146:1016–28. 21925322 10.1016/j.cell.2011.08.008 PMC3176443

[115] Sabari BR, Zhang D, Allis CD, Zhao Y. Metabolic regulation of gene expression through histone acylations. Nat Rev Mol Cell Biol. 2017;18:90–101. 27924077 10.1038/nrm.2016.140 PMC5320945

[116] Andronikou C, Rottenberg S. Studying PAR-dependent chromatin remodeling to tackle PARPi resistance. Trends Mol Med. 2021;27:630–42. 34030964 10.1016/j.molmed.2021.04.010

[117] Henkel R, Hajimohammad M, Stalf T, Hoogendijk C, Mehnert C, Menkveld R, et al. Influence of deoxyribonucleic acid damage on fertilization and pregnancy. Fertil Steril. 2004;81:965–72. 15066449 10.1016/j.fertnstert.2003.09.044

[118] Chai S, Kang J, Wu T, Zheng Y, Zhou X, Xu S, et al. Coevolution and adaptation of transition nuclear proteins and protamines in naturally ascrotal mammals support the Black Queen Hypothesis. Genome Biol Evol. 2024. 10.1093/gbe/evae260. 39688669 10.1093/gbe/evae260 PMC11652718

[119] Leggio L, Paternò G, Cavallaro F, Falcone M, Vivarelli S, Manna C, et al. Sperm epigenetics and sperm RNAs as drivers of male infertility: truth or myth? Mol Cell Biochem. 2025;480:659–82. 38717684 10.1007/s11010-024-04962-w PMC11835981

[120] Shirley CR, Hayashi S, Mounsey S, Yanagimachi R, Meistrich ML. Abnormalities and reduced reproductive potential of sperm from Tnp1- and Tnp2-null double mutant mice. Biol Reprod. 2004;71:1220–9. 10.1095/biolreprod.104.029363. 15189834 10.1095/biolreprod.104.029363

[121] Merges GE, Meier J, Schneider S, Kruse A, Fröbius AC, Kirfel G, et al. Loss of Prm1 leads to defective chromatin protamination, impaired PRM2 processing, reduced sperm motility and subfertility in male mice. Development. 2022. 10.1242/dev.200330. 35608054 10.1242/dev.200330 PMC9270976

[122] Mishra LN, Gupta N, Rao SMR. Mapping of post-translational modifications of spermatid-specific linker histone H1-like protein, HILS1. J Proteome. 2015;128:218–30. 10.1016/j.jprot.2015.08.001 26257145

[123] Arévalo L, Esther Merges G, Schneider S, Schorle H. Protamines: lessons learned from mouse models. Reproduction. 2022;164:R57–74. 35900356 10.1530/REP-22-0107

[124] Wang Y, van Merwyk L, Tönsing K, Walhorn V, Anselmetti D, Fernàndez-Busquets X. Biophysical characterization of the association of histones with single-stranded DNA. Biochimica et Biophysica Acta (BBA). 2017;1861:2739–49. 10.1016/j.bbagen.2017.07.018 28756274

[125] García-Vázquez F, Gadea J, Matás C, Holt W. Importance of sperm morphology during sperm transport and fertilization in mammals. Asian J Androl. 2016;18:844–50. 27624988 10.4103/1008-682X.186880 PMC5109874

[126] Oliva R. Protamines and male infertility. Hum Reprod Update. 2006;12:417–35. 10.1093/humupd/dml009. 16581810 10.1093/humupd/dml009

[127] Moritz L, Schon SB, Rabbani M, Sheng Y, Pendlebury DF, Agrawal R et al. Single residue substitution in Protamine 1 disrupts sperm genome packaging and embryonic development in mice. BioRxiv. 2021;1–32.

[128] Itoh K, Kondoh G, Miyachi H, Sugai M, Kaneko Y, Kitano S, et al. Dephosphorylation of protamine 2 at serine 56 is crucial for murine sperm maturation in vivo. Sci Signal. 2019. 10.1126/scisignal.aao7232. 30914484 10.1126/scisignal.aao7232

[129] Wu JY, Ribar TJ, Cummings DE, Burton KA, McKnight GS, Means AR. Spermiogenesis and exchange of basic nuclear proteins are impaired in male germ cells lacking Camk4. Nat Genet. 2000;25:448–52. 10932193 10.1038/78153

[130] Conrad M, Moreno SG, Sinowatz F, Ursini F, Kölle S, Roveri A, et al. The nuclear form of phospholipid hydroperoxide glutathione peroxidase is a protein thiol peroxidase contributing to sperm chromatin stability. Mol Cell Biol. 2005;25:7637–44. 16107710 10.1128/MCB.25.17.7637-7644.2005 PMC1190272

[131] McLay D. Remodelling the paternal chromatin at fertilization in mammals. Reproduction. 2003;125:625–33. 12713425 10.1530/rep.0.1250625 PMC5123868

[132] Okada Y, Yamaguchi K. Epigenetic modifications and reprogramming in paternal pronucleus: sperm, preimplantation embryo, and beyond. Cell Mol Life Sci. 2017;74:1957–67. 28050628 10.1007/s00018-016-2447-z PMC11107594

[133] Liu Y, Xu Z, Shi J, Zhang Y, Yang S, Chen Q, et al. DNA methyltransferases are complementary in maintaining DNA methylation in embryonic stem cells. iScience. 2022. 10.1016/j.isci.2022.105003. 36117996 10.1016/j.isci.2022.105003 PMC9478929

[134] Alexander KA, Wang X, Shibata M, Clark AG, García-García MJ. TRIM28 controls genomic imprinting through distinct mechanisms during and after early genome-wide reprogramming. Cell Rep. 2015;13:1194–205. 26527006 10.1016/j.celrep.2015.09.078 PMC4644443

[135] Yu M, Liu Y, Han Z, Du W, Chen B, Zhang L, et al. Involvement of PGC7 and UHRF1 in the regulation of DNA methylation of the IG-DMR in the imprinted Dlk1-Dio3 locus. Acta Biochim Biophys Sin (Shanghai). 2022;54:917–30. 35866604 10.3724/abbs.2022080 PMC9828313

[136] Nguyen HT, Dang-Nguyen TQ, Somfai T, Men NT, Beck-Woerner B, Viet Linh N, et al. Excess polyspermy reduces the ability of porcine oocytes to promote male pronuclear formation after in vitro fertilization. Anim Sci J. 2021. 10.1111/asj.13650. 34697861 10.1111/asj.13650 PMC9286444

[137] Markert J, Zhou K, Luger K. Smarcad1 is an ATP-dependent histone octamer exchange factor with de novo nucleosome assembly activity. Sci Adv. 2021. 10.1126/sciadv.abk2380. 34652950 10.1126/sciadv.abk2380 PMC8519567

[138] He M, Zhang T, Yang Y, Wang C. Mechanisms of oocyte maturation and related epigenetic regulation. Front Cell Dev Biol. 2021. 10.3389/fcell.2021.654028. 33842483 10.3389/fcell.2021.654028 PMC8025927

[139] Chen M, Zhu Q, Li C, Kou X, Zhao Y, Li Y, et al. Chromatin architecture reorganization in murine somatic cell nuclear transfer embryos. Nat Commun. 2020. 10.1038/s41467-020-15607-z. 32286279 10.1038/s41467-020-15607-z PMC7156422

[140] Chioccarelli T, Pierantoni R, Manfrevola F, Porreca V, Fasano S, Chianese R, et al. Histone post-translational modifications and circRNAs in mouse and human spermatozoa: potential epigenetic marks to assess human sperm quality. J Clin Med. 2020. 10.3390/jcm9030640. 32121034 10.3390/jcm9030640 PMC7141194

[141] Wang T, Gao H, Li W, Liu C. Essential role of histone replacement and modifications in male fertility. Front Genet. 2019. 10.3389/fgene.2019.00962. 31649732 10.3389/fgene.2019.00962 PMC6792021

[142] Wang J, Gu H, Lin H, Chi T. Essential roles of the chromatin remodeling factor BRG1 in spermatogenesis in mice. Biol Reprod. 2012;86:186. 22495890 10.1095/biolreprod.111.097097 PMC3386149

[143] Zuo X, Rong B, Li L, Lv R, Lan F, Tong MH. The histone methyltransferase SETD2 is required for expression of acrosin-binding protein 1 and protamines and essential for spermiogenesis in mice. J Biol Chem. 2018;293:9188–97. 29716999 10.1074/jbc.RA118.002851 PMC6005419

[145] Firouzabadi, A.M., 2024. Ceratonia Siliqua L: A Natural Compound with Big Impact on Male Reproductive System. Am. J. Mens. Health 18, 15579883241290836. 10.1177/15579883241290835 10.1177/15579883241290835 PMC11504079 39434540

[146] Firouzabadi, A.M., Imani, M., Tofighi Niaki, M., Fesahat, F., 2024. Modulation of NRF2 and CYP24A1 Pathways by Hookah Smoke: Implications for Male Reproductive Health. Am. J. Mens. Health 18. 10.1177/15579883241306893 10.1177/15579883241306893 PMC11645737 39673488

[147] Ghasemzadeh J, Talebi AR, Khalili MA, Fesahat F, Halvaei I, Nabi A, Ashourzadeh S. Sperm parameters, protamine deficiency, and apoptosis in total globozoospermia. Iran J Reprod Med. 2015 Aug;13(8):495-502. PMID: 26568752; PMCID: PMC4637114. PMC4637114 26568752

[148] Firouzabadi, A.R., Firouzabadi, A.M., Shayesteh, M.R., 2025. The Impact of Cadmium Telluride Quantum Dots on Male Reproductive Health: A Systematic Review of Toxicological Effects and Mechanisms. World J. Mens. Health. 10.5534/wjmh.250107 10.5534/wjmh.250107 PMC13302974 40676887

